# An innovative framework to determine the implementation level of personalized medicine: A systematic review

**DOI:** 10.3389/fpubh.2023.1039688

**Published:** 2023-02-03

**Authors:** Lorena Aguilera-Cobos, Patricia García-Sanz, María Piedad Rosario-Lozano, M. Gonzalo Claros, Juan Antonio Blasco-Amaro

**Affiliations:** ^1^Health Technology Assessment Area-AETSA, Andalusian Public Foundation Progress and Health-FPS, Seville, Spain; ^2^Department of Molecular Biology and Biochemistry, Universidad de Málaga, Málaga, Spain; ^3^Institute of Biomedical Research in Málaga (IBIMA), Málaga, Spain; ^4^Centro de Investigación Biomédica en Red de Enfermedades Raras (CIBERER), Instituto de Salud Carlos III, Málaga, Spain; ^5^Institute for Mediterranean and Subtropical Horticulture “La Mayora”, Universidad de Málaga-Consejo Superior de Investigaciones Científicas (IHSM-UMA-CSIC), Málaga, Spain

**Keywords:** personalized medicine (PM), health policy, health system, implementation, framework

## Abstract

**Background:**

Personalized medicine (PM) is now the new frontier in patient care. The application of this new paradigm extends to various pathologies and different patient care phases, such as diagnosis and treatment. Translating biotechnological advances to clinical routine means adapting health services at all levels is necessary.

**Purpose:**

This article aims to identify the elements for devising a framework that will allow the level of PM implementation in the country under study to be quantitatively and qualitatively assessed and that can be used as a guideline for future implementation plans.

**Methods:**

A systematic review was conducted per *the Preferred Reporting Items for Systematic Reviews and Meta-Analyses (PRISMA) statement*. The research question was: What are the domains for determining the level of implementation of PM at the national level? The domains for assessing the degree of PM implementation, which would form the framework, were established.

**Results:**

19 full-text studies that met the inclusion criteria were peer-selected in the systematic review. From all the studies that were included, 37 elements—encompassed in 11 domains—were extracted for determining the degree of PM implementation. These domains and their constituent elements comprise the qualitative and quantitative assessment framework presented herein. Each of the elements can be assessed individually. On the other hand, the domains were standardized to all have the same weight in an overall assessment.

**Conclusions:**

A framework has been developed that takes a multi-factorial approach to determine the degree of implementation of PM at the national level. This framework could also be used to rank countries and their implementation strategies according to the score they receive in the application of the latter. It could also be used as a guide for developing future national PM implementation strategies.

**Systematic review registration:**

https://www.crd.york.ac.uk/prospero/display_record.php?ID=CRD42022338611, Identifier: CRD42022338611.

## 1. Introduction

Great strides have been made in health over the past few decades, resulting in various interventions that have increased the effectiveness and efficiency of healthcare. Thanks to the combination of continuous (bio)technological developments and the need for patient-centered decision-making, medicine has entered an era where greater personalization is possible. Within this context, the term personalized medicine (PM) has arisen. PM is defined as an approach aimed at the prevention, diagnosing, and treating disease based on an individual's specific profile, i.e., taking into account the genetic heterogeneity among individuals, the environment, and their lifestyle (1). PM is a new frontier in healthcare that combines omics, big data analytics, and population health (2). In this study, PM should be understood as an umbrella term for stratified medicine and precision medicine. The authors decided to employ the initialism PM because it is widely used in the scientific literature (3).

PM represents a complete paradigm shift in healthcare thanks to incorporating new diagnosis strategies and allowing new treatments for a wide range of pathologies. In this context, omics play an essential part in PM development (4). Omics are a family of technologies that study biological or molecular elements whose analysis affords a better understanding of the pathophysiology and contextualization of diseases, thus permitting their diagnosis and prevention and/or the application of the correct treatment based on individual differences (5). There are as many omics as there are biological or molecular elements that can be studied using these technologies. They are generally named by adding the suffix “-omics” to the set of molecules and elements studied. Omics technologies should be combined according to the pathology and related to the patient's phenotype. This requires algorithms that integrate both types of data, in addition to other possible patient data such as comorbidities, lifestyle or patient preferences (6, 7). These algorithms can currently be developed thanks to artificial intelligence and machine learning. In addition, data collection can benefit from the use of wearable smart sensors (8).

Genomics is the omics that is leading the implementation in health systems (5, 9). Despite tremendous technological and scientific advances in the medical field, there are significant obstacles to the incorporation of PM into clinical routine. First, collecting genomic and molecular data in healthcare for research purposes is still rare. Secondly, in most cases, clinical and genomic databases are neither homogenized nor interoperable, which hinders progress in understanding diseases. Lastly, the results of data analyses are not always included in the clinical decision-making process or are not efficiently included (10–13).

Health professionals should be trained and motivated to overcome these obstacles using PM and understand its value. Moreover, it would be advantageous if patients, relatives, and the general public were aware of the medical importance of these innovations. Indeed, this awareness-raising should be based on a dialogue between stakeholders to achieve acceptance of implementation (e.g., clinicians, patients/citizens, administration, policymakers). Thus, the full implementation of PM poses significant ethical, legal, regulatory, organizational, and knowledge challenges (4, 14, 15).

In short, a holistic approach to health systems is recommended to achieve the implementation of PM and omics (16). To achieve this, some countries have devised PM implementation strategies. The goal of these strategies is to come up with implementation plans that have a holistic view of patient care and to establish collaborative networks of experts and entities that operate under common standards and protocols (17).

The primary purpose hereof is to develop a framework for determining the level of implementation of PM at the national level based on identifying elements through a systematic review (SR). This framework will allow the degree of implementation to be qualitatively and quantitatively assessed. Additionally, it will be possible to use it as the basis for devising future PM implementation strategies.

## 2. Methods

The development of the framework for determining the level of implementation of PM at the national level was based on the results of a systematic review. This systematic review was conducted per *the Preferred Reporting Items for Systematic Review and Meta-Analyses (PRISMA) statement* (18). The protocol for this review was registered in the PROSPERO repository with ID no CRD42022338611.

### 2.1. Research question

What are the domains for determining the level of implementation of PM at the national level?

### 2.2. Search strategy

For reviewing the scientific evidence, a literature search was carried out (date of search: 14 February 2022) in the following databases of reference: *Medline* (Ovid), *Embase* (Excerpta Medica Database), *WoS* (SCI Science Citation Index), and *PubMed* (*Ahead of print/First online*).

Both controlled language (descriptors) and free terminology (genomic service, personalized medicine, health national program) were used to search for studies, the initial strategy having been adapted to each database's syntax. These searches were limited by date to studies conducted after 2016. The strategies used are listed in Supplementary material.

Likewise, a secondary search was also performed based on the references of the included studies. In addition, the identification of the studies was complemented with a search through the INAHTA database to detect reports from international health technology assessment agencies and a search through institutional or governmental resources for national health policy documents, strategies and regulations.

### 2.3. Selection of the studies to be included

The references identified during the primary and secondary searches in the databases mentioned above were imported into the reference management section of the software application *Covidence* (https://www.covidence.org/), where duplicate references were then identified and deleted. Two authors (LAC, PGS) independently reviewed them to filter out the remaining references by title and abstract using pre-established inclusion and exclusion criteria. Subsequently, both reviewers (LAC, PGS) filtered the full-text studies independently according to the same criteria. Any discrepancies- in both rounds were resolved by both reviewers by consensus.

Technical documents, implementation projects, scientific publications, and regulations dealing with the implementation of PM at the national level and/or its assessment were included. Studies that did not have this purpose were left out, and comments, editorials, review protocols, and clinical trials were excluded by design. Studies published in either English or Spanish after 2016 were included.

### 2.4. Data extraction

The data were extracted by two independent authors (LAC, PGS). First, the authors conducted a descriptive analysis to examine and report the existing methodological frameworks and their characteristics. To this end, general information was extracted from the studies included in the report (authors, year of publication, type of study, features according to study type, and purpose of the study). Secondly, the authors performed a thematic analysis according to the method described by Thomas and Harden (19). Following this methodology, two independent authors (LAC, PGS) identified and extracted the items considered in each of the studies reviewed and included. The classification into domains was based on the list of these items retrieved from the thematic analysis, by a consensus process. After this classification, the items acquired the status of assessable elements of the framework.

### 2.5. Quality of the studies included

The quality of the studies included was assessed independently by two authors (LAC, PGS), which resolved any disagreements they had by consensus. Different quality assessment tools were used depending on the type of study. The AMSTAR-II tool (20) was used to assess the quality of systematic reviews; the SANRA tool (21) was used to assess the quality of narrative reviews, and the checklist published by Humphrey-Murto (22) was used to assess the quality of interviews and expert panels.

### 2.6. Development of a framework for determining the level of personalized medicine implementation

The items extracted from the included studies in the SR were used to develop a framework for the qualitative and quantitative determination of PM implementation at the national level. It should be noted that the exclusion criteria were not based on the number of studies in which these items appeared. The authors used the items extracted through the thematic analysis (19) of the selected studies to generate the framework. Then, the authors grouped these items into domains to generate a comprehensible and functional tool. Following this classification, the items acquired the status of assessable elements of the framework. These domains were developed *ad-hoc*, and their definitions and the elements they contained were established by the authors using a consensus methodology. These elements were individually assessable and quantifiable with a simple scoring system. This simple approach to assigning points across the different domains was based on the idea that the value of each domain was the same according to the contained elements. This assumption enables standardizing all domains' weight in the final score. This scoring system allows the implementation level to be quantitatively determined and is based on similar quantitative assessment tools (23, 24).

## 3. Results

**Figure 1:**
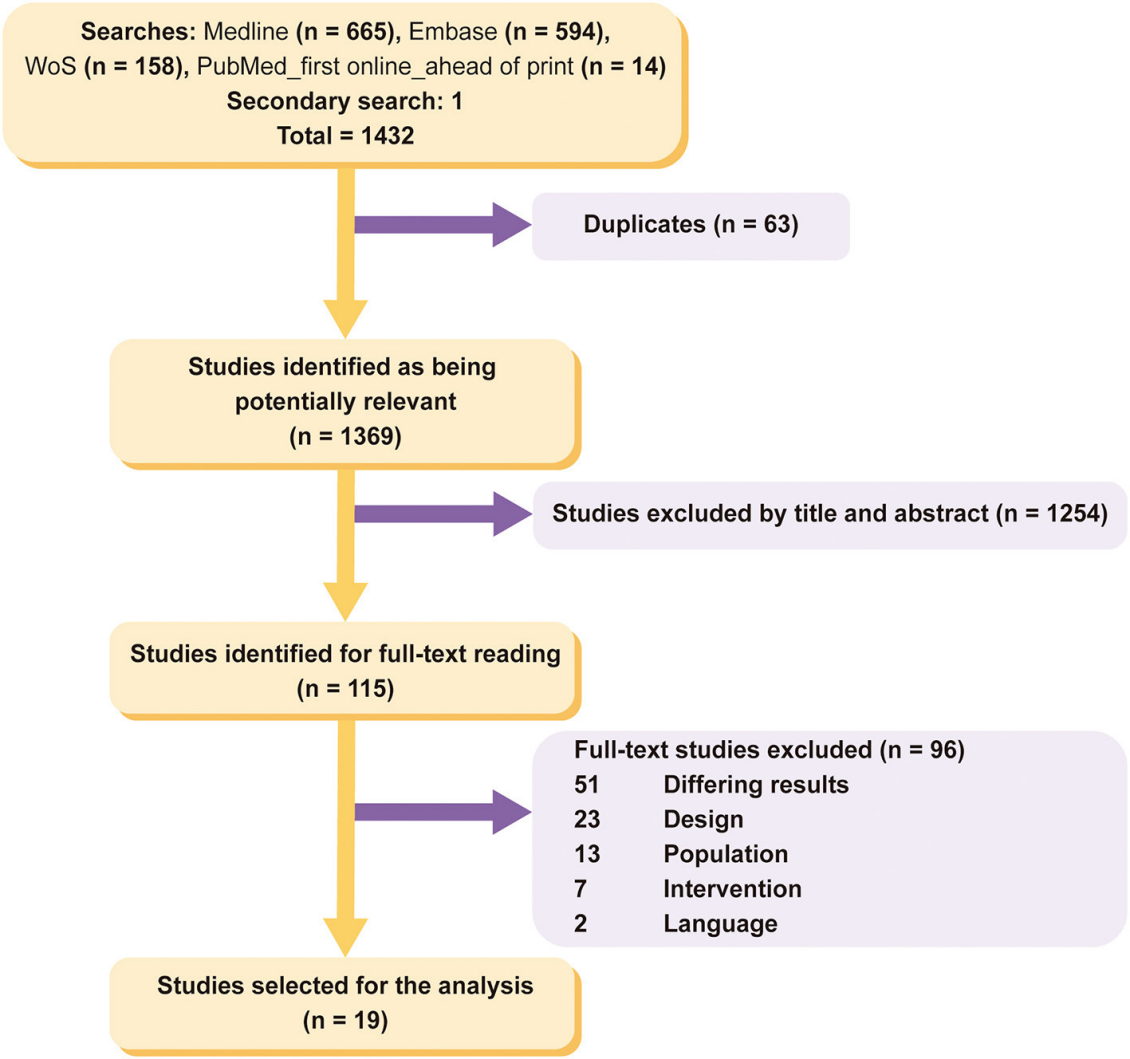
PRISMA flowchart.

A total of 1,432 studies were found in the initial search. After eliminating duplicates, 1,369 potentially relevant studies were left. The studies were independently filtered by title and abstract by both authors, yielding 115 potentially relevant studies. After the peers filtered the studies by full text, 19 full-text studies were ultimately included (11, 12, 23–39). It should be noted that only one study was found that met the inclusion criteria from the secondary and complementary research (24). This filtering process is shown in the PRISMA flowchart in Figure 1.

### 3.1. Description of the studies included

The information about the description of the studies included in this review was extracted by two independent authors as described in Methods. Tables listing these characteristics, divided by type of study, are shown in Supplementary Tables S1–S3. Those reviews that also included interviews or an expert panel are listed in the corresponding two tables. It is worth noting that some of the narrative reviews included lacked a methodology, which prevented the authors from extracting the information they contained.

### 3.2. Assessment of the quality of the studies included

The quality of the studies included was assessed using different tools according to the type of study: AMSTARII (20) for systematic reviews, SANRA (21) for narrative reviews, and the checklist proposed by Humphrey-Murto (22) for interviews and expert panels. In the case of the reviews that also included interviews or expert panels, both elements were assessed separately.

The quality of each of the studies included is listed in Supplementary Table S4. According to the criteria of the AMSTARII tool, the quality of the systematic reviews was rated moderate, low, or critically low; none of the reviews included in the SR had the high quality that is the top level in this tool. According to the SANRA tool's criteria, the quality of the narrative reviews ranged from 12 to 5, 12 being the maximum possible score in this tool. The authors of the SANRA tool (21) suggest that a score of 4 or lower indicates the inferior quality of the review in question. The quality of the interviews and expert panels scored, according to the checklist published by Humphrey-Murto, between 11 and 3, 11 being the maximum possible score in this tool. No studies were ultimately excluded on low-quality grounds.

### 3.3. Domains for determining the level of PM implementation

**Figure 2:**
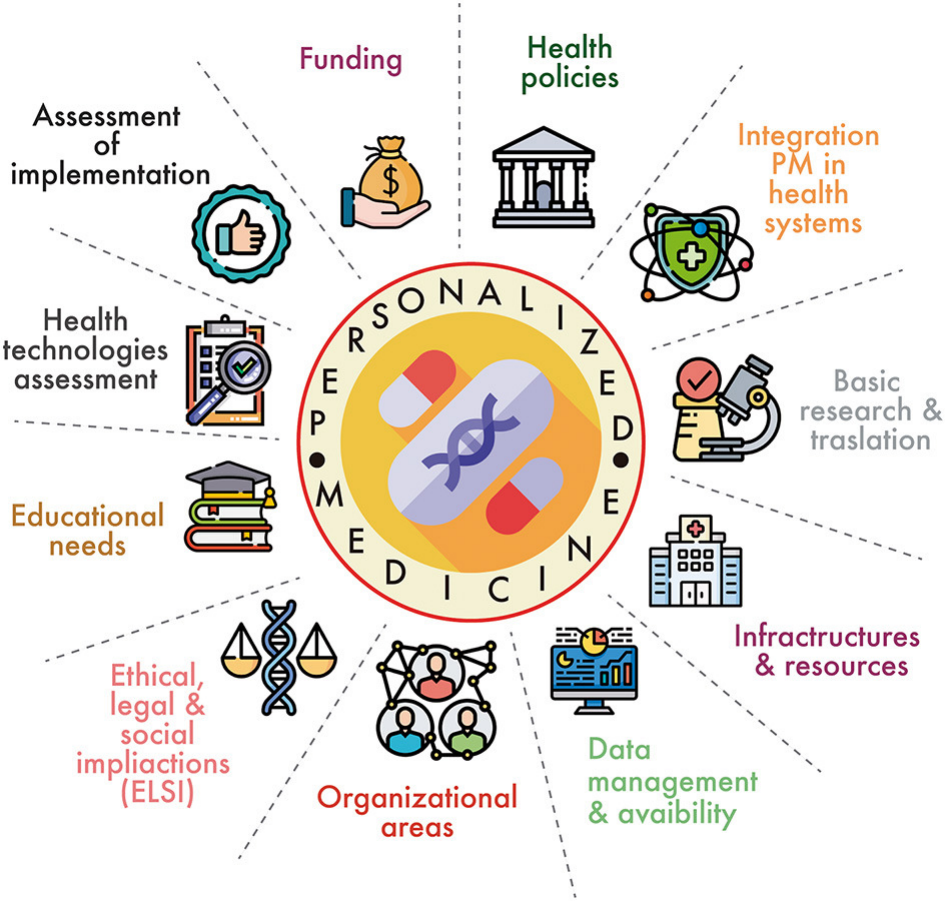
Graphical representation of framework domains. Source: Flaticon.com.

The elements for determining the level of implementation of PM at the national level were extracted from the studies included in the SR via a thematic analysis and after classification into domains through a consensus process. Figure 2 represents these domains graphically. Each of these domains contains several quantifiable elements. Table 1 lists all domains and the scoring elements for each domain. The domains and the assessable elements of which they are composed are described below. The references cited in each domain name refer to the studies where their assessable elements were identified. The results of the extraction of the included studies to obtain the assessable elements that constitute the framework can be found in Supplementary Table S5.

**Table 1:** Framework domains and elements for determining the level of PM implementation.

**Health policies**
• Setting in motion toward legislative measures (i.e., existing project or initiative to reach this aim) • Legislation/regulation • Collaborative working groups among the different stakeholders
**Integration of PM in the health system**
• Portfolio of PM services • Level of accessibility of PM in the health system • Portfolio of purpose of care
**Basic research and translation**
• Plan for the promotion of basic research in PM and translation to clinical research
**Infrastructures and resources**
• Projects for gathering omics information at the population level • Biobanks (biological samples at the population or pathology-specific level) • Electronic health record storage platforms • Omics data storage platforms • Omics data and big data analysis platforms
**Data management and availability**
• Harmonization, quality, and protection of electronic health records • Harmonization, quality, and protection of omics data • Incorporation of omics data into electronic health records • Access to omics data of and interoperability among practitioners and entities • Use of omics data in clinical decision-making
**Organizational areas**
• Interoperability among basic and translational research organizations and resources • Introduction of areas specializing in PM • Reinforcement of non-specialized areas involved in PM • Development and adoption of procedural guidelines • Organizational structure of omics testing • Omic testing equipment
**Ethical, legal, and social implications (ELSI)**
• Patient information care level • Standardized patient informed consent forms and/or the patient acceptance and commitment • Data protection mechanisms
**Educational needs**
• Education and training for healthcare staff specialized and non-specialized in PM • Education of patients and relatives in PM • Awareness-raising and outreach activities for the citizenry
**Assessment of health technologies**
• PM-specific health technology assessment plan • Health technology assessment body • PM-specific health technology assessment methodology • HTA decision-making group
**Assessment of implementation**
• PM implementation evaluation plan • Implementation evaluation body • Implementation evaluation methodology
**Funding**
• PM implementation budget forecast

#### 3.3.1. Health policies

This domain refers to the setting in motion of legislative measures that will provide an adequate legal framework for patients to access to PM with the utmost guarantees. This domain also gathers the possibility that the legislative rules never materialize, for that reason, includes the following elements: Setting in motion toward legislative measures (i.e., existing project or initiative), the existence of specific legislation or regulations for one or more areas of PM, and the creation of legislative working groups for laying down these rules or devising projects or initiatives are included in this domain as valuable elements (23, 27, 31, 33, 38).

#### 3.3.2. Integration of PM in the health system

This domain envisages the incorporation of PM and omics into the corresponding national health system. The quantifiable elements included in this domain are the existence of a portfolio of PM services, a high level of accessibility to PM for patients in the health system, and the collection of the care purposes for which the use of PM is being considered (23, 34, 36–39).

#### 3.3.3. Basic and translational research

This domain contains a single scorable element: the existence of a plan for promoting basic and translational research in PM (30, 31, 38).

#### 3.3.4. Infrastructures and resources

This domain deals with gathering samples and clinical data at the population or pathology-specific level and their proper storage and analysis. This domain includes five scorable elements: the existence of projects for gathering omics information at the population level; the existence or setting up of biobanks for depositing and preserving biological samples; and the existence of platforms for storing and analyzing electronic medical records and omics data (24, 29, 37–39).

#### 3.3.5. Data management and availability

This domain considers the specific needs at the level of the data generated in omics tests and PM so that they may be used effectively in clinical decision-making. Five scorable elements were considered to assess this domain: harmonization, quality, and protection of electronic health records and omics data; incorporation of omics data into electronic health records; data access by and interoperability among practitioners and organizations; and use of omics data in clinical decision-making (11, 24, 28–30, 34, 37, 38).

#### 3.3.6. Organizational areas

Changes are required at the organizational level after considering PM to be a change in the approach to medicine. These changes arise from a need for specialization of the health system and staff and are included in the six measurable elements this domain comprises: interoperability among organizations and research resources; implementation of health areas specializing in PM; reinforcement of non-specialized health areas involved in PM; development and adoption of procedures that include PM; setting up of organizational structures for omics testing; and the existence of omic testing equipment (12, 23–25, 27, 29, 31, 34, 37, 39).

#### 3.3.7. Ethical, legal, and social implications (ELSI)

This domain refers to the fact that it is essential to develop PM implementation strategies within an ethical and legal framework that boosts potential health benefits while minimizing potential damages, such as misuse of information, stigmatization, or discrimination. To this end, patients need to be adequately informed and their PM data legally protected, given its sensitivity, the potential impact of the results on their health, and the likelihood of accidental findings in some tests. Thus, this domain includes the following assessable elements: The existence of standardized patient informed consent forms and/or the patient acceptance and commitment prior to testing; data and results in protection mechanisms; standardization of the level of care in charge of patient information (11, 25, 26, 29, 38).

#### 3.3.8. Educational needs

Given that PM will be a major innovation for health staff, patients, relatives, and society, it is essential that they be adequately trained in how to use it and made aware of when it is being used. This is a complex issue and refers to the so-called public engagement. Hence, efficient and successful engagement entails a coordinated strategy and organizational effort across various fields, from public health to science and education. This domain includes three elements to cover all educational needs: the existence of educational plans for patients and families in PM; the existence of educational plans for patients and families in PM; and the existence of awareness-raising and outreach activities for the citizenry (social awareness and citizens' omics science literacy) (26, 27, 29, 31, 38).

#### 3.3.9. Health technologies assessment (HTA)

The health technologies used in omics testing have peculiarities that require adapting the HTA methodology. Four elements have been included to ensure that these peculiarities do not become an obstacle to the assessment of these technologies and, hence, to their implementation: the existence of a specific assessment plan for HTA in PM; the creation of a reference body for the HTA of PM technologies; the specific adaptation of an HTA methodology to PM; and the creation of a group of experts for HTA decision-making (35, 37, 38).

#### 3.3.10. Implementation assessment

The current boom in implementing PM-related technologies calls for devising implementation evaluation plans for monitoring it and identifying areas for improvement. The elements considered in this domain were: the existence of a plan for evaluating the implementation of PM at the national level; the existence of a competent body; and the existence of an appropriate evaluation methodology (25, 31, 33, 36, 37).

#### 3.3.11. Funding

The creation and development of all the domains above must be adequately funded. Only one scorable element was considered to assess this domain: the existence of a funding plan for the implementation of PM (25, 31).

### 3.4. Framework for the qualitative and quantitative determination of personalized medicine implementation level

The domains proposed here for determining the level of PM implementation and their assessable elements extracted from the studies included in the systematic review allowed us to develop a framework. This framework allows this level of implementation at the national level to be qualitatively and quantitatively determined and is designed to be applicable to any country and health system.

This tool includes 11 domains comprising 37 individually quantifiable elements in all. If a national implementation plan does not include an element or no information about it can be found, it will be given a score of 0. If a national implementation plan includes an element, it will be given a score of 1. If an element is already in the implementation phase, it will be given a score of 2.

Based on two of the studies included, (23, 24) a mathematical formula was applied to standardize the weight of the domains in the final assessment of the implementation. In such a way, those domains with a higher number of assessable elements do not have a bigger weight in the implementation's final score. A spreadsheet for automatically making these calculations is included in the Supplementary material.

When applying the framework to compare or rank countries, consideration should be given to whether it was possible to gather and collect enough information from each country. Otherwise, the benchmarking may not represent the actual situation of each health system compared.

## 4. Discussion

A multidisciplinary team of experts in this kind of study and PM conducted a systematic review as part of this study and identified the elements needed to develop a new framework following a thematic analysis and consensus methodology. This proposed framework will allow the degree of PM implementation in different international health systems to be qualitatively and quantitatively determined.

Our research question and systematic review provide a comprehensive approach to implementing a PM that considers the many interrelationships existing in today's health systems. In particular, we devised a literature search strategy with a holistic approach to correctly implementing PM in health systems. The basic elements we identified in other frameworks, models or studies served as a basis for developing a framework. The framework we developed allows the different aspects of PM implementation in various countries and their strengths and weaknesses to be qualitatively and quantitatively assessed. This framework may also be used at the health policy strategy level and for planning the implementation of PM.

To our knowledge, this is the first time a framework for determining the country-level implementation of PM in different countries has been proposed and described with a holistic approach in health systems. Some of the studies we considered when developing our framework include partial tools for assessing PM implementation in specific organizational areas and applications (12, 23, 32). Agarwal et al. (23) focus on assessing the integration of PM in US health organizations. Doyle et al. (32) solely focus on the organizational aspects of the centers providing PM. Lee et al. (12) developed a global investment innovation framework with regional, technical and organizational dimensions to lay the foundations for a global, national PM strategy, but it focused only on South Korea.

Another original aspect of our framework stems from an SR conducted by a multidisciplinary team of experts in this type of study and PM. This SR allowed us to identify the domains for determining the level of PM implementation and the assessable elements of which they are composed. The studies we included in the SR have different authors from different countries, which allows us to assert that it will be possible to use our framework in different countries. We extracted these domains and their elements from the studies we included in the SR, which we discussed in the results section and in this section below. In summary, the included studies we reviewed highlighted critical themes from our findings that endorse the domains of the proposed framework for determining the level of PM implementation.

The European Health Systems & Policies report, and the WHO report indicate that health policies and policymakers need to consider concrete, change-driven actions to strengthen health systems and enhance their performance (40). In this way, health systems can adapt to change and improvement. This means that any effort to improve the performance of health systems must be developed through policy implementation challenges (41). Because of this and the holistic changes required for PM implementation, health policy is one of the primary keys to the actual and full implementation of PM in health systems. In fact, health policy is one of the domains of our framework that encompasses three elements from five included studies (23, 27, 31, 33, 38). This domain focuses on setting legislative measures in motion that will provide an adequate legal framework for patients to access PM with the utmost guarantees. Ultimately, this domain also depicts potential transformative strategies and methods for defining and measuring value at all decision-making levels aligned with PM, where the collaborative working groups among the different stakeholders also play an essential role (42). Regarding this, a well-coordinated health policy facilitates PM integration into healthcare systems (39). This integration is contemplated in our framework with a domain composed of three elements drawn from six of the included studies (23, 34, 36–39). Indeed, patient access to PM is variable since there is no uniform standard for integrating PM into healthcare systems (43). Proper and standardized integration will allow the PM to be applied in appropriate delivering points of care and health care purposes. To carry out this integration, all stakeholders, their needs, and benefits must be taken into account (32).

Promoting basic and translational science in PM favors the bidirectional flow of development and implementation between the laboratory and clinical practice, which is essential for PM implementation. This comprises a domain with a single assessable element drawn from three of the included studies (30, 31, 38). This promotion should include long-term actions due to the long development times of the scientific studies until their incorporation into clinical routine (44).

The necessary and adequate resources must be available for PM proper implementation. The framework includes five elements from five studies (24, 30, 37–39) related to these needs. Some of these needs imply the reinforcement of existing resources and infrastructures, and others imply the creation of new structures. These resources are essential for the storage, preservation, and accessibility of biological samples and electronic patient data, as well as for their adequate analyze (45). It also identified as essential in implementing PM the need for standardized digital systems, remarkably reliable information formats, and digital decision support tools in electronic health records (27, 46, 47). The growing PM data must not only be stored or analyzed, but also managed in a specific way that ensures interoperability, standardization, and security. Some of the characteristics of these data are specific, e.g., their large volume, high sensitivity, and need for interoperability, and would therefore require specific actions (48). These necessities have been considered in our framework, which includes five elements extracted from eight of the included studies (11, 24, 28–30, 34, 37, 38) about this issue. Proper PM data management and safety would hasten their availability in clinical practice and increase patient confidence in the healthcare system. Health systems and their organizational areas must be understood as living systems adaptable to change and improvement. This need for adaptation becomes imperative in the case of PM implementation, which requires system-wide adaptations. This adaptation appears in 10 of the included studies (12, 23–25, 27, 29, 31, 34, 37, 39), from which the six elements that constitute the domain “Organizational areas” were extracted. Therefore, adaptations of organizational areas should not only be understood as the creation of new areas but also as the strengthening of pre-existing areas and the relationships between them (49).

PM implementation's success depends increasingly on its competence to improve healthcare for all population groups and citizens' commitment (50). The ethical, legal, and social implications of PM emerge as an essential key to addressing this aim due to the sensitivity of the data generated linked to PM application, the potential impact of the results on health, and the likelihood of accidental findings. Indeed, the ethical, legal, and social implications have an identity as a domain in our framework, whose elements were extracted from five included studies (11, 25, 26, 29, 38). This domain considers correct patient information and engagement based on standardized protection and information mechanisms. Although PM progresses rapidly, the problem of the need for more ethical, legal, and social regulations remains. There should be regulation on the return of results, confidentiality, and privacy that should be carried out by both policymakers and legislators together. In addition, they should encourage policies that promote PM education and fund initiatives that bring together the interests of different stakeholders (50, 51). Hence, the existence of specific legislation or initiatives toward this end, bioethics committees, and society's awareness of PM would facilitate PM implementation (52). Evidence points out that public awareness about PM could be higher due to a lack of trust and trustworthiness, which are essential in supporting the acceptance of the PM. There must be mechanisms to enhance this trust and trustworthiness via promoting transparency about the social value of PM, the correct use of the new data generated, and the consequence of ethical issues (46). In this context emerges the need for public engagement in PM. Public engagement is a complex theme that encompasses the commitment and acceptance of society at large and the patients, families, and groups most directly affected by the diseases addressed by PM. Therefore, there is no doubt about the importance of education and awareness raising in PM for its proper implementation. This education should be tailored to stakeholders (policymakers, scientists, clinicians, patients, or citizens). Awareness-raising must be based on a dialogue between stakeholders to achieve PM acceptance (51), being able to lead by the government or different organizations already actively engaging the public (46). These differential educational needs according to the target audience are reflected in the domain “Educational needs” of our framework, whose elements have been extracted from five included studies (26, 27, 29, 31, 38).

The increasing growth of medical innovation, especially in PM, creates a need to improve patient involvement in the health technology assessment (HTA) process. Therefore, the methodology for HTA needs to be adapted to the particularities of technologies used in PM. The lack of standardized methods due to different national policies and cultural disparities has led to different recommendations for the same healthcare technology (43). Indeed, the new European HTA legislation is developing to solve this and prioritizes assessing some of the leading health technologies in PM, such as advanced therapies (53, 54). HTA agencies have acquired a key role in assessing and approving these new technologies related to PM. Expert and multidisciplinary teams from these agencies should carry out these assessments and their adaptation process. Three of the included studies (35, 37, 38) consider the three elements necessary for this assessment that is included in the framework. Agencies and stakeholders involved in HTA should work together to elevate the patient voice in HTA worldwide in a creative and transformative way.

As we have seen so far, several mechanisms are necessary for implementing PM, so evaluating the correct functioning and development of each of them in all phases of implementation is necessary. This evaluation should be done by specialized teams and with appropriate methodological evaluation tools (4). This will allow the detection of possible shortcomings or barriers in the implementation and suggest possible improvements. The assessment of the implementation domain that encompasses these features in our framework contains three assessable elements from five studies (25, 31, 33, 36, 37). Funding is another essential domain to ensure that all the domains mentioned above work and interact appropriately in a stable, long-term, and secure manner. This assessment element is addressed in two of the included studies (25, 31). Such project-based funding across different PM domains will provide the necessary support for PM from the research phase to its implementation in the clinical routine.

As far as the study's limitations are concerned, a possible publication bias in the detection of studies is worth noting, despite having conducted a search in other sources (such as governmental websites) of information in addition to the primary search. This could be one of the reasons why the studies detected that met the inclusion criteria were less numerous. It must be considered that the assessment of PM commitment and patient acceptance has yet to develop by a specific methodology fully. Hence, this element will be more complex to identify. Another limitation is that this study does not directly include the opinion of experts; nevertheless, studies that contain expert opinions have been included. Moreover, it may be necessary to successively update this study, given the continuous evolution and development of PM. We expect to publish a report on the application of the results of this study in the future.

The framework we have discussed herein permits a quantitative determination of the level of PM implementation thanks to the standardized scoring system we have developed. This system assigns equal weight to all domains in the final score. The adoption of this system is warranted by different studies (23, 39) suggesting that for the PM to be properly implemented, it must be understood as a gear in which all its parts interact and are equally important to its operation. The scores chosen (0, 1, and 2) allow those countries that are in the process of taking measures to be rewarded, it is understood that the complexity of some measures may cause them to take longer to implement in practice. A similar scoring methodology was developed in Agarwal et al.'s framework (23). Our tool penalizes studies with a score of 0 when no information can be found on any of the elements. This penalty can spur the countries concerned to do a better job of publishing and raising awareness of their national PM implementation strategies.

Since this is a quantitative framework, countries could be tentatively ranked or sorted, for guidance purposes, based on their levels of PM implementation. Such ranking could allow the highest-scoring national initiatives to be detected. Additionally, it might be useful to identify them as models or benchmarks that could then be used by other countries to come up with their own PM implementation initiatives in the future.

Something that has become clear after conducting the systematic review of studies and extracting therefrom the domains on which this framework is based is that the different stakeholders need to work together. In other words, for the PM to be properly implemented in different countries, the process needs to include different types of profiles, such as legislators, scientists, health professionals, patients, and different public and private entities. The involvement and collaboration of an educated, aware society are necessary for the implementation process to be successful. In view of these numerous collaborations and interrelationships, plans for articulating these initiatives should be drawn up. These plans should encompass all profiles and consider all implementation domains for which strong and stable funding is essential.

It is advisable for each country to evaluate its own PM implementation plan so that it may understand its strengths and weaknesses. This could allow preventative solutions to be found and new domains of action detected. Determining the level of PM implementation may be an ongoing process, given the rate of progress of both basic science and translational science in this field. In light of the many areas, professionals, and patients that have a bearing on PM, this determination should be understood as a PM-specific health system performance assessment (HSPA).

The correct implementation of PM in current health environments poses a major challenge in that the concept of PM encompasses many kinds of basic sciences, clinical specialties, pathologies, and patients. Thus, it will cause a paradigm shift in current medicine that will necessitate reforming the national health systems. This is why this study aims at a holistic approach to properly implementing PM in these health systems. It should also be taken into consideration, however, that the development of PM and its initial application must take place within each country's regulatory framework.

## 5. Conclusions

This study develops and proposes the first framework for the qualitative and quantitative determination of the degree of PM implementation at the international level. This innovative framework is based on a systematic review and can be used by countries with a high level of PM implementation, countries in the process of implementing PM, and countries interested in embarking on this implementation process. The adaptability of this framework to different health systems and its methodological rigor give it additional value in dealing with an emergent field with so much potential to benefit the stakeholders involved in implementing PM (e.g., scientists, clinicians, policymakers, patients, citizens). This framework, which includes a broad collection of domains obtained from a systematic review of studies, ensures PM will have a holistic and integrative approach within the different national health systems.

## Data availability statement

The original contributions presented in the study are included in the article/Supplementary material, further inquiries can be directed to the corresponding authors.

## Author contributions

LAC and PGS: conceptualization, methodology, collecting data, data extraction and analysis, interpreting the results, drafting, editing, and reviewing the manuscript. MPRL: methodology, design, and the bibliographic strategy and search. MGC: reviewing the manuscript. JABA: coordination, interpreting the results, reviewing the manuscript, and financial support. All authors have reviewed and approved the manuscript's contents and the authorship requirements have been met.
