# Supplementary material for: An innovative framework to determine the implementation level of personalized medicine: A systematic review
Source: Front Public Health. 2023 Feb 3;11:1039688. doi: 10.3389/fpubh.2023.1039688 (PMC9936069; doi:10.3389/fpubh.2023.1039688)
Supplement: Supplementary file 2 [file Table_2.DOCX]

Supplementary Material

# Supplementary Data

- **Search strategies**

Medline (OVID)

1 exp Genomics/ (135052)

2 exp Genetic Testing/ (51057)

3 ((gene or genetic or genom* or multigene or multi-gene) adj3 (test* or panel*)).tw. (58499)

4 Genetic Services/ or Genetic Counseling/ (15402)

5 (genetic adj3 (service or counsel* or screening?)).tw. (29322)

6 (genomic? adj3 (resource or service? or portfolio)).tw. (1316)

7 (Prenatal adj3 (Noninvasive or Screening)).tw. (5527)

8 ((mass or carrier) and screening).tw. (42895)

9 *molecular diagnostic techniques/ (7905)

10 ((molecular or genetic) and techni*).tw. (157471)

11 Pharmacogenetics/ (12868)

12 (pharmacogenomic$ or pharmacogenetic$).ti,ab. (17398)

13 Precision Medicine/ (23193)

14 (((individualized or personalized or precision or predictive or stratified) adj3 medicine) or 'p health' or p-health or theranostic or theranostics).tw. (35431)

15 1 or 2 or 3 or 4 or 5 or 6 or 7 or 8 or 9 or 10 or 11 or 12 or 13 or 14 (506540)

16 Health Plan Implementation/ or Translational Medical Research/ (18521)

17 (implement* or disseminat$ or translation or transfer or incorporation or integration).tw. (1653569)

18 16 or 17 (1663276)

19 Health Resources/ (14527)

20 National Health Programs/ (33072)

21 ((health adj3 (national or program? or insurance)) or "GOVERNMENT PROGRAMS").ti. (39614)

22 Regional Health Planning/ (5454)

23 ((health adj3 planning) or (annual adj3 plan?) or (Appropriateness adj3 Review)).tw. (13725)

24 Health Systems Plans/ (194)

25 (Health adj3 Plan?).tw. (19681)

26 Program Development/ (30160)

27 (program adj3 (development or description?)).tw. (9740)

28 (strategy adj3 (national or plan)).tw. (3864)

29 Health Policy/ (70488)

30 (Polic* adj3 (Health or Healthcare)).ti,ab. (52908)

31 "Delivery of Health Care"/ (104818)

32 (Delivery adj3 (Health or Healthcare)).tw. (28823)

33 Health Services Accessibility/ (82457)

34 (Access* adj3 (care or Services or Therap* or treartment?)).tw. (59767)

35 Community Health Services/og, st [Organization & Administration, Standards] (10550)

36 (Community adj3 Service?).tw. (18435)

37 19 or 20 or 21 or 22 or 23 or 24 or 25 or 26 or 27 or 28 or 29 or 30 or 31 or 32 or 33 or 34 or 35 or 36 (483316)

38 15 and 18 and 37 (1241)

39 animals/ not (animals/ and humans/) (4924998)

40 38 not 39 (1235)

41 limit 40 to yr="2016 -Current" (665)

**Embase**

**594**

**#40**

**#39** AND [embase]/lim NOT ([embase]/lim AND [medline]/lim) AND [2016-2022]/py AND [humans]/lim

**2,981**

**#39**

**#15** AND **#19** AND **#38**

**809,453**

**#38**

**#20** OR **#21** OR **#22** OR **#23** OR **#24** OR **#25** OR **#26** OR **#27** OR **#28** OR **#29** OR **#30** OR **#31** OR **#32** OR **#33** OR **#34** OR **#35** OR **#36** OR **#37**

**16,622**

**#37**

(**community** NEAR/3 **service?**):ti,ab

**10,637**

**#36**

**'community care'**/de AND **'organization and management'**/de

**80,490**

**#35**

(**access*** NEAR/3 (**care** OR **services** OR **therap*** OR **treartment?**)):ti,ab

**73,462**

**#34**

**'health care access'**/de

**34,770**

**#33**

(**delivery** NEAR/3 (**health** OR **healthcare**)):ti,ab

**193,477**

**#32**

**'health care delivery'**/de

**61,732**

**#31**

(**polic*** NEAR/3 (**health** OR **healthcare**)):ti,ab

**207,582**

**#30**

**'health care policy'**/de

**5,350**

**#29**

(**strategy** NEAR/3 (**national** OR **plan**)):ti,ab

**12,346**

**#28**

(**program** NEAR/3 (**development** OR **description?**)):ti,ab

**25,279**

**#27**

**'program development'**/de

**11,852**

**#26**

(**health** NEAR/3 **plan?**):ti,ab

**12**

**#25**

**'health systems plans'**:ti,ab

**16,066**

**#24**

((**health** NEAR/3 **planning**):ti,ab) OR ((**annual** NEAR/3 **plan?**):ti,ab) OR ((**appropriateness** NEAR/3 **review**):ti,ab)

**70**

**#23**

**'regional health planning'**:ti,ab

**38,517**

**#22**

((**health** NEAR/3 (**national** OR **program?** OR **insurance**)):ti) OR **'government programs'**:ti

**216,521**

**#21**

**'public health'**/de

**7,080**

**#20**

**'health resources'**:ti,ab

**2,106,575**

**#19**

**#16** OR **#17** OR **#18**

**2,005,263**

**#18**

**implement***:ti,ab OR **disseminat***:ti,ab OR **translation**:ti,ab OR **transfer**:ti,ab OR **incorporation**:ti,ab OR **integration**:ti,ab

**127,416**

**#17**

**'health care planning'**/exp OR **'translational research'**/exp

**55**

**#16**

(**'health plan'** NEAR/3 **implementation**):ti,ab

**645,628**

**#15**

**#1** OR **#2** OR **#3** OR **#4** OR **#5** OR **#6** OR **#7** OR **#8** OR **#9** OR **#10** OR **#11** OR **#12** OR **#13** OR **#14**

**47,430**

**#14**

(((**individualized** OR **personalized** OR **precision** OR **predictive** OR **stratified**) NEAR/3 **medicine**):ti,ab) OR **'p health'**:ti,ab OR **theranostic**:ti,ab OR **theranostics**:ti,ab

**56,978**

**#13**

**'personalized medicine'**/de

**25,311**

**#12**

**pharmacogenomic***:ti,ab OR **pharmacogenetic***:ti,ab

**21,303**

**#11**

**'pharmacogenetics'**/de

**192,357**

**#10**

(**molecular**:ti,ab OR **genetic**:ti,ab) AND **techni***:ti,ab

**6,874**

**#9**

**'molecular diagnosis'**/mj

**60,752**

**#8**

(**mass**:ti,ab OR **carrier**:ti,ab) AND **screening**:ti,ab

**9,439**

**#7**

(**prenatal** NEAR/3 (**noninvasive** OR **screening**)):ti,ab

**411**

**#6**

(**genomic?** NEAR/3 (**resource** OR **service?** OR **portfolio**)):ti,ab

**30,830**

**#5**

(**genetic** NEAR/3 (**service** OR **counsel*** OR **screening?**)):ti,ab

**36,406**

**#4**

**'genetic service'**/de OR **'genetic counseling'**/de

**94,709**

**#3**

((**gene** OR **genetic** OR **genom*** OR **multigene** OR **'multi gene'**) NEAR/3 (**test*** OR **panel***)):ti,ab

**103,617**

**#2**

**'genetic screening'**/exp

**120,990**

**#1**

**'genomics'**/exp

**Web Of Science (WOS)**

61

**#60 AND #42 AND #39**

[15](https://www-webofscience-com.bvsspa.idm.oclc.org/wos/alldb/summary/4dd4ef67-8823-466e-83d3-9e878694e647-249ada68/relevance/1)8

60

**#59 OR #58 OR #57 OR #56 OR #55 OR #54 OR #53 OR #52 OR #51 OR #50 OR #49 OR #48 OR #47 OR #46 OR #45 OR #44 OR #43**

[339,311](https://www-webofscience-com.bvsspa.idm.oclc.org/wos/alldb/summary/9ec95f40-b367-4284-9a20-070121c091a6-249ada61/relevance/1)

59

**TI=(Community NEAR/3 Service?) OR AB=(Community NEAR/3 Service?)**

[20,156](https://www-webofscience-com.bvsspa.idm.oclc.org/wos/alldb/summary/d6454f0f-b6da-4310-90de-5adb9b04de6b-249a79a5/relevance/1)

58

**TI=("Health Services organization")**

[22](https://www-webofscience-com.bvsspa.idm.oclc.org/wos/alldb/summary/8261038e-8dbf-46eb-be3e-aea8d1d4f10a-249a7682/relevance/1)

57

**TI= ("access care" or "access services" or "access therapy" or "access treartment") OR AB=("access care" or "access services" or "access therapy" or "access treartment")**

[2,147](https://www-webofscience-com.bvsspa.idm.oclc.org/wos/alldb/summary/26e9e8d0-bc28-483e-8bfe-68ab83025983-249ada53/relevance/1)

56

**TI=("Health Services Accessibility") OR AB=("Health Services Accessibility")**

[57](https://www-webofscience-com.bvsspa.idm.oclc.org/wos/alldb/summary/9e4c50ca-3dbf-410e-b012-3cd065eadb33-249a711b/relevance/1)

55

**TI=("Delivery Health" or "Delivery Healthcare") OR AB=("Delivery Health" or "Delivery Healthcare")**

[224](https://www-webofscience-com.bvsspa.idm.oclc.org/wos/alldb/summary/82d54234-096d-44ab-a8dc-b5dcab00e9dd-249ac8af/relevance/1)

54

**TI="Delivery of Health Care" OR AB="Delivery of Health Care"**

[1,499](https://www-webofscience-com.bvsspa.idm.oclc.org/wos/alldb/summary/a3ede24f-f1ab-44ef-b74d-c81cd02d868a-249a6b19/relevance/1)

53

**TI=(Polic* Health" or "Polic* Healthcare") OR AB=(Polic* Health" or "Polic* Healthcare")**

[4](https://www-webofscience-com.bvsspa.idm.oclc.org/wos/alldb/summary/052ae671-d8cd-4cd8-99cf-87bb254a2035-249acd59/relevance/1)

52

**TI="Health Policy" OR AB="Health Policy"**

[22,925](https://www-webofscience-com.bvsspa.idm.oclc.org/wos/alldb/summary/5cefd11d-4efa-4109-88f8-7978e44185ea-249a66f7/relevance/1)

51

**TI=( "national strategy " or "national plan") OR AB=( "national strategy " or "national plan")**

[4,184](https://www-webofscience-com.bvsspa.idm.oclc.org/wos/alldb/summary/581605fe-6508-45df-8f65-a42f9079aecf-249a6478/relevance/1)

50

**TI=(program NEAR/3 development or description?) OR AB=(program NEAR/3 development or description?)**

[193,836](https://www-webofscience-com.bvsspa.idm.oclc.org/wos/alldb/summary/130c9b5e-4fb5-40f3-abf9-ec8c9c027073-249a632c/relevance/1)

49

**TI="Program Development" OR AB="Program Development"**

[5,238](https://www-webofscience-com.bvsspa.idm.oclc.org/wos/alldb/summary/a4561220-8afa-4e6c-9d9d-d7bbdce5cbb9-249a6101/relevance/1)

48

**TI=(Health NEAR/3 Plan?) OR AB=(Health NEAR/3 Plan?)**

[13,107](https://www-webofscience-com.bvsspa.idm.oclc.org/wos/alldb/summary/4a56e027-0493-439a-823f-ea9cde0b0be0-249a5fb7/relevance/1)

47

**TI="Health Systems Plans" OR AB="Health Systems Plans"**

[4](https://www-webofscience-com.bvsspa.idm.oclc.org/wos/alldb/summary/827753f0-f3be-4062-9704-b836c3db4e0a-249a5ec6/relevance/1)

46

**TI=(health NEAR/3 planning) or TI=(annual NEAR/3 plan?) or TI=(Appropriateness NEAR/3 Review) OR AB=(health NEAR/3 planning) or AB=(annual NEAR/3 plan?) or AB=(Appropriateness NEAR/3 Review)**

[34,473](https://www-webofscience-com.bvsspa.idm.oclc.org/wos/alldb/summary/4379b572-0be3-4d10-8db9-3a7bfa49ebd6-249a5df3/relevance/1)

45

**TI=("Regional Health Planning") OR AB=("Regional Health Planning")**

[45](https://www-webofscience-com.bvsspa.idm.oclc.org/wos/alldb/summary/71a8bf7f-0885-4d2b-bb04-fa0ea42f3212-249a5d09/relevance/1)

44

**TI=("health national" or "health program?" or "health insurance" OR "GOVERNMENT PROGRAMS") OR AB=("health national" or "health program?" or "health insurance" OR "GOVERNMENT PROGRAMS")**

[56,344](https://www-webofscience-com.bvsspa.idm.oclc.org/wos/alldb/summary/667876d5-6bd8-4e69-9963-7b2ade4940ce-249a5c2f/relevance/1)

43

**TI=("Health Resources" OR "National Health Programs") OR AB=("Health Resources" OR "National Health Programs")**

[5,285](https://www-webofscience-com.bvsspa.idm.oclc.org/wos/alldb/summary/653f045e-54ed-4544-a0b0-17683cb060d5-249a5ac0/relevance/1)

42

**#41 OR #40**

[1,208,620](https://www-webofscience-com.bvsspa.idm.oclc.org/wos/alldb/summary/a4fd10a1-d657-427d-8867-6a637a2d2a54-249a85b3/relevance/1)

41

**TI=(implementation or dissemination or translation or transfer or incorporation or integration) OR AK=(implementation or dissemination or translation or transfer or incorporation or integration)**

[1,208,553](https://www-webofscience-com.bvsspa.idm.oclc.org/wos/alldb/summary/a024ff2b-784a-4894-8233-c44e870b1227-249a85ab/relevance/1)

40

**TI=("Health Plan Implementation" or "Translational Medical Research") or AB=("Health Plan Implementation" or "Translational Medical Research")**

[74](https://www-webofscience-com.bvsspa.idm.oclc.org/wos/alldb/summary/2ab62309-793d-4c55-ab64-71ea27dce555-249a55b9/relevance/1)

39

**#30 OR #31 OR #32 OR #33 OR #34 OR #35 OR #36 OR #37 OR #38**

[1,090,964](https://www-webofscience-com.bvsspa.idm.oclc.org/wos/alldb/summary/bd61ca9d-1d5a-46b5-bf9b-8b04d15c1db1-249a54bb/relevance/1)

38

**TI=("individualized medicine" or "personalized medicine" or "precision medicine" or "predictive medicine" or "stratified medicine")**

[10,328](https://www-webofscience-com.bvsspa.idm.oclc.org/wos/alldb/summary/14611e76-7366-46b0-a6c5-f01be28a8495-249a52b5/relevance/1)

37

**TI=("Precision Medicine") OR AB=("Precision Medicine")**

[14,061](https://www-webofscience-com.bvsspa.idm.oclc.org/wos/alldb/summary/728f8e43-3ca7-4745-85c5-3ca0b3cb2386-249a51c6/relevance/1)

36

**TI=(pharmacogenomic or pharmacogenetic) OR AB=(pharmacogenomic or pharmacogenetic)**

[11,072](https://www-webofscience-com.bvsspa.idm.oclc.org/wos/alldb/summary/97a1b83d-feea-41fe-b75b-7ad987e9354a-249a5066/relevance/1)

35

**TI=("molecular diagnostic techniques") OR AB=("molecular diagnostic techniques")**

[474](https://www-webofscience-com.bvsspa.idm.oclc.org/wos/alldb/summary/ff5aeec6-383d-4cec-a1af-fb6984be4fef-249a4f52/relevance/1)

34

**TI=("genomic resource" or "genomic service" or "genomic portfolio") OR AB=("genomic resource" or "genomic service" or "genomic portfolio")**

[622](https://www-webofscience-com.bvsspa.idm.oclc.org/wos/alldb/summary/21f51bf6-ddf3-46e6-8fd5-f7cefdb870c7-249a4da2/relevance/1)

33

**TI=(genetic NEAR/3 service or counsel* or screening?) OR AB=(genetic NEAR/3 service or counsel* or screening?)**

[161,271](https://www-webofscience-com.bvsspa.idm.oclc.org/wos/alldb/summary/80bb49e8-f24b-459f-ae12-82b2d2799a9e-249a4cb4/relevance/1)

32

**TI=(gene AND test* or panel*) OR TI=( genetic AND test* or panel*) OR TI=( genome AND test* or panel*) OR TI=(multiline AND test* or panel*) OR TI=( multi-gene AND test* or panel*) OR AB=(gene AND test* or panel*) OR AB=( genetic AND test* or panel*) OR AB=( genome AND test* or panel*) OR AB=(multiline AND test* or panel*) OR AB=( multi-gene AND test* or panel*)**

[859,521](https://www-webofscience-com.bvsspa.idm.oclc.org/wos/alldb/summary/50b33071-7d1d-4626-afe0-f55e9bdc6bd7-249a4b97/relevance/1)

31

**TI=("Genetic Services" or Genetic Counseling") or AB=("Genetic Services" or Genetic Counseling")**

[364](https://www-webofscience-com.bvsspa.idm.oclc.org/wos/alldb/summary/52adb7d1-cee2-453c-9aef-cdfb46a3b30e-249a4a37/relevance/1)

30

**TI=(Genomics OR "Genetic Testing") OR AB=(Genomics OR "Genetic Testing")**

[87,802](https://www-webofscience-com.bvsspa.idm.oclc.org/wos/alldb/summary/59c4279b-028f-4838-9ced-e5ab74e95d7d-249a4759/relevance/1)

# Supplementary Figures and Tables

| Table S1. Description of the systematic reviews included | | | | |
| --- | --- | --- | --- | --- |
| Author and year | **Databases and search date** | **Selection criteria** | **Number of studies included** | **Purpose** |
| Kovanda et al.^24^ 2021 | PubMed, Google, and the European Genome-Phenome Archive (EGA).  April 2020. | Projects for the implementation of genomic medicine at the national level.  Projects that had already been completed as of the date of the search, international projects and projects that only focused on obtaining sequencing samples were excluded. | 41 studies from 86 countries included | To provide an overview of available information on ongoing national genomics projects worldwide to identify the features they had in common and the differences among them. |
| Pearce et al.^32^ 2019 | Web of Science, and PubMed.  April 2018. | The review included primary research studies and systematic reviews. The search was not limited to UK studies nor to countries with similar health systems.  Articles not written in English were excluded.  Editorials, comments, conference abstracts and methodological papers that did not discuss the research results were excluded. Articles with a molecular/biological focus, papers on perinatal, prenatal-perinatal or prenatal genetics, and articles on genetic research were also excluded. | 55 studies included  (USA: 29; UK: 10; Canada: 4; Netherlands: 2; Estonia: 1; South Korea: 1; Cuba: 1) | To identify the critical domains for introducing genomic medicine into clinical practice and assess the current state of the UK’s National Health Service at implementing a Genomic Medicine Service. |
| Pitini et al.^33^ 2019 | PubMed, Scopus, ISI Web of Knowledge, Google Scholar, and Google, and the websites of major government agencies and research organisations taking part in the evaluation of genetic testing.  April 2017. | Any document describing an original evaluation framework for genetic testing. | 29 studies included | To identify and compare existing genomic test evaluation frameworks, focusing on their evaluation criteria (analytical and clinical validity, clinical utility). |
| Unim et al.^34^ 2019 | PubMed, Scopus, Web of Science, Google, and Google Scholar.  2015 | The following were included: Relevant articles and reports on pilot studies, best practices and funded projects intrinsic to the provision of genetic services, the provision of all sorts of genetic testing by teams of geneticists and primary or secondary healthcare professionals, and interventions in European and non-European (English-speaking) countries (USA, Canada, Australia, and New Zealand).  Studies published in English and Italian between 2000 and 2015 were included.  Studies that only informed of genetic advising services, descriptive studies where care channels were not well defined, and studies that did not specify the type of considered genetic test were excluded. | 117 studies included | To evaluate the genetic services in European and non-European (English-speaking) countries to identify and rank genetic testing implementation models. |

| Table S2. Description of the narrative reviews included | | |
| --- | --- | --- |
| Author and year | **Methodological features of the study** | **Purpose** |
| Bilkey et al.^35^  2019 | - | They pose different stages of the development cycle to describe genomic healthcare applications, their scope, and the ethical, legal and societal challenges. |
| Burns et al.^36^  2019 | - | To arrive at key considerations for successfully integrating genomic technologies into health systems. To describe the effective, sustainable implementation of genomic testing in the strategic priority areas of Australia’s National Health Genomics Policy Framework. |
| Chong et al.^37^  2018 | 47 resources included (38 articles, 6 guides from government agencies, and 3 websites).  Databases: PubMed, Embase, CINAHL, Cochrane Library, Web of Science, and grey literature.  Date of search: 31 Jan 2017  Countries included: Indonesia, Malaysia, Singapore, and Thailand. | To describe the current approaches of the policies and programmes in Southeast Asia to promoting the adoption and application of personalised medicine. To highlight the challenges and propose strategies for future development. |
| Doyle et al.^38^  2018 | Drafting in an interactive-process working group comprising 8 members of the Genomics and Population Health Action Collaborative. 38 measures for the implementation of genomic medicine were identified. | To develop an organisational framework for the implementation of genomic services through the identification of broad domains of state programmes of public health genomics (with emphasis on Lynch syndrome, hereditary colon cancer, and hereditary breast and ovarian cancer). These implementation measures will be part of a set of genomic public health tools that will be available online. |
| Kurnat-Thoma^39^ 2020 | 80 resources included (51 articles, and 29 online resources).  Databases: PubMed, CINAHL, and Google Scholar.  MeSH terms: Clinical Laboratory Improvement Amendments (CLIA)-certified laboratory genetic tests, health system implementation, genetic test implementation, ELSI, precision medicine/precision health genomic medicine implementation, workforce development, and clinical decision support. | To describe current trends (2014-2019) in the US in the use of genetic testing in the health system, of resources for education and training of the health staff in genetics/genomics, and of tools for the implementation of genomic medicine into clinical practice. |
| Lee & Kim^12^  2022 | 5,647 R&D projects related to personalised medicine included.  Databases: National Institute of Science and Technology, South Korea. | To develop a precise global innovation framework with regional, technical and organisational dimensions in order to establish a collaboration among the different stakeholders, thus laying the foundations for a global national personalised medicine strategy in South Korea. |
| Pastorino et al.^25^ 2021 | - | To describe the ethical issues raised by the implementation of personalised medicine, how these issues may affect its efficacy or efficiency, and mechanisms for mitigating these effects. |
| Vrijenhoek et al.^26^  2021 | - | To provide an overview of the genetic services offered in Estonia, Finland, and the Netherlands, and to consider the impact of historical and recent events on the development of clinical genetics. To describe the key characteristics of the genomic services in these 3 countries and identify similarities and differences among these systems at the cultural, structural and clinical practice levels and how they affect their future development. |
| Whitsel et al.^27^  2019 | - | To highlight the significant role the US government, at its different organisational levels, will play in the different stages of development of personalised medicine (from basic science to translation). |

| Table S3. Description of other types of studies included (surveys, questionnaires, interviews, discussions, and expert panels) | | |
| --- | --- | --- |
| Author and year | **Methodological features of the study** | **Purpose** |
| Agarwal et al.^23^  2021 | Structured survey to 4 leading US health organisations in PM: two community health systems, one distribution network, and one academic health system. The interviews were conducted according to a double-blind format. The names of the institutions that were interviewed are not disclosed. | To find out how health systems have adopted project management, understand the challenges associated with the clinical integration of project management, and develop a perspective on the extent of the adoption of project management at these different institutions. These interviews served as the basis for identifying which parameters the institutions at the forefront of project management value and the key parameters according to which these organisations measure the level of project management adoption. From these interviews, eight framework assessment criteria were defined. Three of these criteria pertained to the tests conducted and the data collected so as to make personalised medicine possible. The remaining categories were test orientation and data accessibility, leadership support, internal funding, data utilisation, and data sharing efforts. |
| Barash^11^  2016 | Survey to 60 attendees at the 11th Asia-Pacific Conference on Human Genetics, 2015, Hanoi, Vietnam.  5-question questionnaire; 2 of the questions were open-ended. | To make a regional consensus statement on the needs to make progress in the implementation of personalised medicine and genomics in clinical practice so as to help funding organisations and policy makers promote the sharing of genomic benefits globally. |
| Chanfrau-Coffinier et al.^28^  2019 | Interviews of 57 attendees at the Veterans Health Administration Conference held on 25 August 2016 in Arlington, Virginia (11 administrators/managers, 22 clinicians, 6 patients/relatives, 5 politicians/payers, 13 researchers). | To develop a model for facilitating the implementation of precision medicine in the Veterans Health Administration and other health organisations. |
| Delnord et al.^29^  2021 | Survey to 14 experts from the ComPerMed Board consisting in a questionnaire made up of 28 questions grouped into 4 categories (quality, responsiveness, stakeholder commitment, and knowledge integration). Scoring system according to Likert scale. Previously provided documentation (NGS Roadbook). | To evaluate the implementation of the NGS Roadbook in the Belgian health system and the impact this initiative had on the use of NGS in oncohematology. |
| Pitini^33^  2019 | Expert panel using the Delphi methodology in 3 rounds; 55 members of the Italian Network of Public Health Genomics (34 physicians, 20 biologists, and 1 economist). | To develop a framework for the comprehensive evaluation of genetic testing that includes the evaluation of service provision. |
| Pritchard et al.^30^  2017 | Survey to the Health Working Group composed of representatives from 49 health service organisations (19 academic health centres, 12 community health systems, 16 health care support organisations, and 2 groups of physicians).  Discussion with 54 participants divided into 3 focus groups (17 suppliers, 23 industry experts, and 14 patients); each were subjected to a round of 5 different questions. | Identification of the health areas requiring improvement for personalised medicine to be implemented and of the strategies to address these improvements primarily analysed in US health organisations. |
| Ricciardi et al.^31^  2017 | Discussion of health, public policy, public health, law and ethics experts from Europe, the US and Canada on the future of healthcare organised by the Foundation for Genomics and Population Health. | To describe the challenges to and the benefits and consequences of the introduction of personalised medicine by policy makers and health staff. |

| Table S4. Assessment of the quality of the studies included | | | |
| --- | --- | --- | --- |
| Author and year | **Type of study** | **Quality tool** | **Quality result** |
| Kovanda et al.^24^ 2021 | Systematic review | AMSTARII | Low quality |
| Pearce et al.^32^ 2019 | Systematic review | AMSTARII | Moderate quality |
| Pitini et al.^33^ 2019 | Systematic review | AMSTARII | Critically low quality |
| Unim et al.^34^ 2019 | Systematic review | AMSTARII | Low quality |
| Bilkey et al.^35^ 2019 | Narrative review | SANRA | 8 |
| Burns et al.^36^ 2019 | Narrative review | SANRA | 7 |
| Chong et al.^37^ 2018 | Narrative review | SANRA | 12 |
| Doyle et al.^38^ 2018 | Narrative review | SANRA | 10 |
| Kurnat-Thoma^39^ 2020 | Narrative review | SANRA | 11 |
| Lee & Kim^12^ 2022 | Narrative review | SANRA | 12 |
| Pastorino et al.^23^ 2021 | Narrative review | SANRA | 5 |
| Vrijenhoek et al.^26^ 2021 | Narrative review | SANRA | 6 |
| Whitsel et al.^27^ 2019 | Narrative review | SANRA | 5 |
| Agarwal et al.^23^ 2021 | Interview | Humphrey-Murto | 8 |
| Barash^11^ 2016 | Survey | Humphrey-Murto | 4 |
| Chanfrau-Coffinier et al.^28^ 2019 | Interview | Humphrey-Murto | 6 |
| Delnord et al.^29^ 2021 | Survey | Humphrey-Murto | 8 |
| Pitini^33^ 2019 | Expert panel | Humphrey-Murto | 11 |
| Pritchard et al.^30^ 2017 | Survey | Humphrey-Murto | 6 |
| Ricciardi et al.^31^ 2017 | Expert panel | Humphrey-Murto | 3 |

| **Table S5. Extraction table of framework elements from the included studies** | |
| --- | --- |
| **Health policies elements** | **Included studies** |
| - Setting in motion towards legislative measures (i.e., existing project or initiative to reach this aim) | Chong et al.^37^,  Vrijenhoek et al.^26^ |
| - Legislation/Regulation | Chong et al.^37^,  Vrijenhoek et al.^26^,  Agarwal et al.^23^,  Ricciardi et al.^31^ |
| - Collaborative working groups among the different stakeholders | Chong et al.^37^,  Agarwal et al.^23^,  Delnord et al.^29^ |
| **Integration of PM in the health system elements** | **Included studies** |
| - Portfolio of PM services | Pearce et al.^32^,  Unim et al.^34^,  Chong et al.^37^,  Agarwal et al.^23^ |
| - Level of accessibility of PM in the health system | Pearce et al.^32^,  Burns et al.^36^,  Chong et al.^37^,  Doyle et al.^38^ |
| - Portfolio of purpose of care | Pearce et al.^32^,  Chong et al.^37^ |
| **Basic research and translation** | **Included studies** |
| - Plan for the promotion of basic research in PM and translation to clinical research | Chong et al.^37^,  Chanfreau-Coffinier et al.^28^,  Delnord et al.^29^ |
| **Infrastructures and resources** | **Included studies** |
| - Projects for gathering omics information at the population level | Doyle et al.^38^,  Barash et al.^11^ |
| - Biobanks | Chong et al.^37^,  Doyle et al.^38^ |
| - Electronic health record storage platforms | Kovanda et al.^24^ |
| - Omics data storage platforms | Burns et al.^36^,  Barash et al.^11^ |
| - Omics data and big data analysis platforms | Doyle et al.^38^,  Barash et al.^11^ |
| **Data management and availability** | **Included studies** |
| - Harmonization, quality, and protection of electronic health records | Pearce et al.^32^,  Chanfreau-Coffinier et al.^28^ |
| - Harmonization, quality, and protection of omics data | Whitsel et al.^27^,  Barash et al.^11^ |
| - Incorporation of omics data into electronic health records | Chong et al.^37^,  Chanfreau-Coffinier et al.^28^ |
| - Access to omics data of and interoperability among practitioners and entities | Kovanda et al.^24^,  Bilkey et al.^35^,  Whitsel et al.^27^ |
| - Use of omics data in clinical decision-making | Kovanda et al.^24^,  Bilkey et al.^35^,  Whitsel et al.^27^ |
| **Organizational areas** | **Included studies** |
| - Interoperability among basic and translational research organizations and resources | Pearce et al.^32^,  Agarwal et al.^23^ |
| - Introduction of areas specializing in PM | Kovanda et al.^24^,  Kurnat-Thoma et al.^39^ |
| - Reinforcement of non-specialized areas involved in PM | Burns et al.^36^,  Kurnat-Thorma et al.^39^,  Lee & Kim^12^,  Vrijenhoek et al.^26^,  Barash et al.^11^ |
| - Development and adoption of procedural guidelines | Kovanda et al.^24^,  Vrijenhoek et al.^26^,  Delnord et al.^29^ |
| - Organizational structure of omics testing | Unim et al.^34^,  Pritchard et al.^30^ |
| - Omic testing equipment | Pritchard et al.^30^ |
| **Ethical, legal and social implications (ELSI)** | **Included studies** |
| - Patient information care level | Chong et al.^37^,  Kurnat-Thoma et al.^39^ |
| - Standardized patient informed consent forms and/or the patient acceptance and commitment | Chong et al.^37^,  Pastorino et al.^25^,  Barash et al.^11^ |
| - Data protection mechanisms | Bilkey et al.^35^ |
| **Educational needs** | **Included studies** |
| - Education and training for healthcare staff specialized and non-specialized in PM | Pastorino et al.^25^,  Barash et al.^11^,  Delnord et al.^29^ |
| - Education of patients and relatives in PM | Pastorino et al.^25^,  Barash et al.^11^ |
| - Awareness-raising and outreach activities for the citizenry | Chong et al.^37^,  Vrijenhoek et al.^26^ |
| **Assessment of health technologies** | **Included studies** |
| - PM-specific health technology assessment plan | Pitini et al.^33^,  Chong et al.^37^ |
| - Health technology assessment body | Pitini et al.^33^,  Chong et al.^37^ |
| - PM-specific health technology assessment methodology | Pitini et al.^33^,  Burns et al.^36^ |
| - HTA decision-making group | Pitini et al.^33^ |
| **Assessment of implementation** | **Included studies** |
| - PM implementation evaluation plan | Unim et al.^34^,  Burns et al.^36^,  Ricciardi et al.^31^ |
| - Implementation evaluation body | Unim et al.^34^,  Kurnat-Thoma et al.^39^ |
| - Implementation evaluation methodology | Unim et al.^34^,  Delnord et al.^29^ |
| **Funding** | **Included studies** |
| - PM implementation budget forecast | Burns et al.^36^,  Delnord et al.^29^ |
